# *Cymbiola nobilis* shell: Toughening mechanisms in a crossed-lamellar structure

**DOI:** 10.1038/srep40043

**Published:** 2017-01-17

**Authors:** Hongmei Ji, Xiaowu Li, Daolun Chen

**Affiliations:** 1 Department of Materials Physics and Chemistry, School of Material Science and Engineering, Northeastern University, Shenyang 110819, China; 2 Department of Mechanical and Industrial Engineering, Ryerson University, 350 Victoria Street, Toronto, Ontario M5B 2K3, Canada

## Abstract

Natural structural materials with intricate hierarchical architectures over several length scales exhibit excellent combinations of strength and toughness. Here we report the mechanical response of a crossed-lamellar structure in *Cymbiola nobilis* shell via stepwise compression tests, focusing on toughening mechanisms. At the lower loads microcracking is developed in the stacked direction, and channel cracking along with uncracked-ligament bridging and aragonite fiber bridging occurs in the tiled direction. At the higher loads the main mechanisms involve cracking deflection in the bridging lamellae in the tiled direction alongside step-like cracking in the stacked direction. A distinctive crack deflection in the form of “convex” paths occurs in alternative lamellae with respect to the channel cracks in the tiled direction. Furthermore, a barb-like interlocking mechanism along with the uneven interfaces in the 1st-order aragonite lamellae is also observed. The unique arrangement of the crossed-lamellar structure provides multiple interfaces which result in a complicated stress field ahead of the crack tip, hence increasing the toughness of shell.

Compared with the contemporary manufacturing technology of humanity, nature has achieved great successes that can provide us with direct clues in designing lighter, stronger and tougher materials^1^,^2^,^3^. Natural structural materials, usually consisting of hard and soft phases that are arranged in intricate hierarchical architectures over several length scales, exhibit remarkable mechanical properties^4^,^5^,^6^,^7^,^8^,^9^,^10^. This is why mimicking the structural features of natural structural materials has become a fascinating and thriving area in recent years^11^,^12^,^13^,^14^,^15^,^16^. For example, Ritchie and co-workers^11^ developed a silicon carbide/polymethyl methacrylate (SiC/PMMA) composite with a laminated structure similar to that of nacre, and they reported that such SiC/PMMA composites can display significantly higher *R*-curve toughness values, indeed higher than any silicon carbide ceramic to date. Therefore, natural biological materials can provide a rich source of inspiration to solve a classic material-design dilemma that the strength and toughness as two key structural properties tend to be mutually exclusive^4^.

Mollusca, as the second largest phylum in nature, can offer a wide range of successful composition design. While extensive studies have been focused on the simpler “brick-and-mortar” shell microstructure named nacre^17^,^18^, there have been limited experimental efforts on the crossed-lamellar structure^19^ although this type of structure consists of more than 90% species within the Mollusca^20^. This structure is hierarchically assembled by ordinary brittle inorganic calcium carbonate with only 0.1–1 wt.% organic matrix^21^,^22^. The crossed-lamellar structure in conch *Strombus gigas* has drawn a lot of attention, and its microstructure has been well described^19^,^21^,^22^,^23^,^24^,^25^,^26^,^27^,^28^. Specifically, the structure is stacked by the 1st-order lamellae, which are further composed of laths (the 2nd-order lamellae) of parallel mineral fibers (the 3rd-order lamellae). The fibers are parallel within a given 1st-order lamella but almost perpendicular to those in the neighboring 1st-order lamellae. The architecture of the three-layer crossed-lamellae structure in conch *Strombus gigas* is in a 0°/90°/0° mode, i.e., the arranged direction of the 1st-order lamellae in the middle layer has a 90° rotation with respect to those in the inner and outer layers. The mechanical tests, including bending, compression and indentation tests^21^,^22^,^23^,^24^,^25^,^26^,^27^,^28^, indicated that this shell fails gradually, i.e., in the form of so-called ‘graceful failure’, reflecting a superior toughness compared with pure mineral aragonite. Actually, the unique construction of the crossed-lamellar structure provides multiply and complex interfaces at different levels of lamellae, which provide several energy dissipating mechanisms during deformation, such as multiple microcracking, crack bridging and crack deflection^6^. Both the experimental results^21^ and finite element modeling^19^ indicated that channel cracking along the 1st-order interfaces in the inner and outer layers and crack bridging through the 2nd-order interfaces of the middle layer can significantly increase the work of fracture of materials. Recently, Shin *et al*.^26^ reported a toughening mechanism governed by nanoscale twins, whose boundaries can effectively impede crack propagation by inducing phase transformation and delocalization of deformation around the crack tip.

Despite some limited studies on the toughening mechanisms of conch *Strombus gigas* shell, there is still a lack of systematic understanding on the toughening mechanisms in the crossed-lamellar structure in *Cymbiola nobilis* shell. It is unclear whether the channel cracking and crack bridging also occur along the specific lamellae interfaces like conch *Strombus gigas* shell, and how different toughening mechanisms act synergistically during deformation. The purpose of this investigation was, therefore, to study the mechanical response of the crossed-lamellar structure in a *C. nobilis* shell under stepwise compressive deformation, focusing on toughening mechanisms. This study will provide a theoretical basis for developing high-performance biomimetic structural materials.

## Results

### Microstructure of *C. nobilis* shell

**Figure 1:**
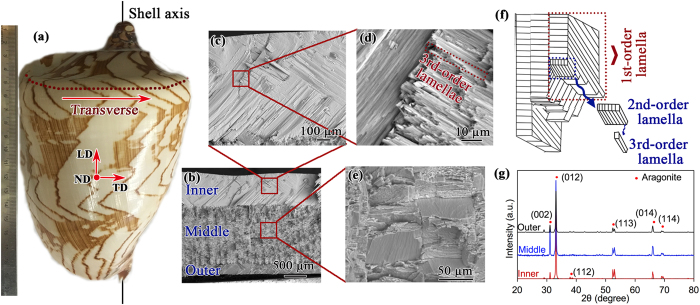
Overall view (**a**) and multi-scale hierarchical structures of *C. nobilis* shell, i.e., (**b**) morphologies on the cross-section of a directly broken sample, (**c**,**d**) detailed features of the crossed-lamellar structure with the tiled 1st-order lamellae in the inner layer, (**e**) detailed features of the crossed-lamellar structure with the stacked 1st-order lamellae in the middle layer, (**f**) schematic drawing of the crossed-lamellar structure, and (**g**) XRD patterns of the inner, middle and outer layers of *C. nobilis* shell. Note that in (**a**) LD indicates the longitudinal direction (shell axis direction), TD stands for the transverse direction, and ND represents the normal direction through the shell thickness; the observational direction is parallel to the LD.

Figure 1 shows the morphology of TD-ND cross section of a directly broken *C. nobilis* shell sample. It is seen that the shell exhibits a 0°/90°/0° mode that three distinct layers consist of a crossed-lamellar structure and the arranged direction of the 1st-order lamellae in the middle layer has a 90° rotation to those in the inner and outer layers, as indicated in Fig. 1(b). The magnified morphologies of this structure are shown in Fig. 1(c–e). It is clearly seen that the crossed-lamellar structure exhibits different morphologies along two nearly vertical directions with respect to the arrangement of the 1st-order lamellae, i.e., one being the tiled direction (Fig. 1(c)), and the other being the stacked direction (Fig. 1(e)). As schematically shown in Fig. 1(f), the lath-like 2nd-order lamellae are parallel within a given 1st-order lamella but almost perpendicular to those in the neighboring 1st-order lamellae, and the 3rd-order fibers are parallel stacked within a lath. XRD analysis reveals that the mineral in each layer is the same, i.e., aragonite calcium carbonate, as shown in Fig. 1(g).

### Compressive behavior

**Figure 2:**
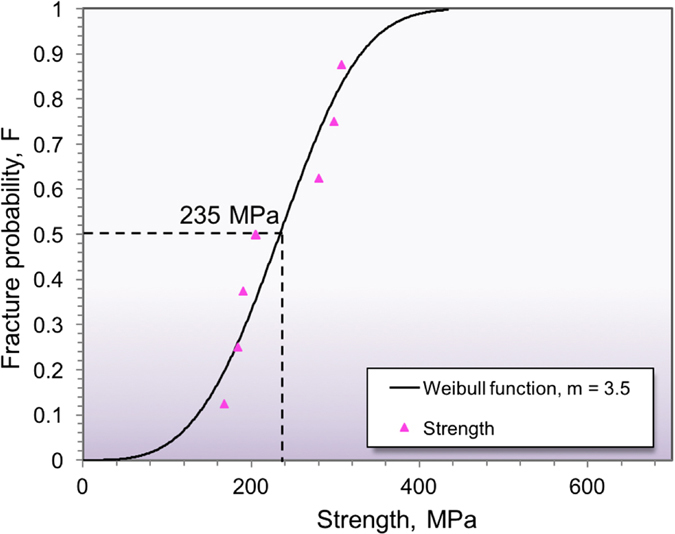
Weibull plot of compressive strength of *C. nobilis* shell samples.

Figure 2 shows the compressive fracture strength of the crossed-lamellar structure along the TD. It is seen that the Weibull distribution function could be used to well characterize the scatter of the distribution of fracture strength, where the strength corresponding to a 50% fracture probability (F(*V*) = 50%) is obtained to be ~235 MPa. Similar Weibull distribution function has also been used to characterize the flexural strength results of biomaterials^29^,^30^. The compressive strength of the present crossed-lamellar structure is much higher than that of many other biomaterials, such as *Aligator osteoderms*^31^ and *Leatherback sea turtle* shells^32^, whose compressive strengths are 40–70 MPa and 10–45 MPa, respectively. In the *Strombus gigas* shell, as being one of the most famous shells, its compressive strength at the 50% fracture probabilities is about 218 MPa along the same direction as the present shell^22^. Thus, the compressive strength is different in a variety of shells even with a similar crossed-lamellar structure, and it is a little higher in the present shell than that in the *Strombus gigas* shell.

To understand the mechanical behavior and examine the detailed fracture characteristics of the crossed-lamellar structure in the present shell, stepwise compression tests were performed on a single sample via a loading-unloading procedure at prescribed loads. After each loading cycle, the initiation and propagation of cracks and the interaction between cracks among three layers were observed on the pre-polished TD-ND cross section called the main observation surface (MOS).

**Figure 3:**
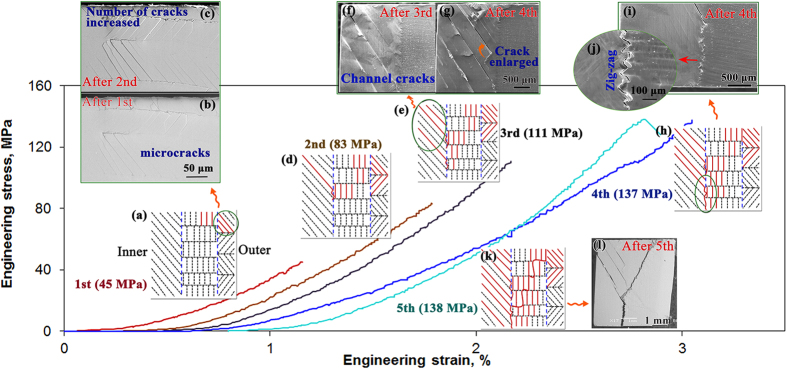
Stepwise compression stress-strain curves and the corresponding surface changes on the MOS after each loading cycle in the *C. nobilis* shell. (**a**,**d**,**e**,**h**,**j**) Schematic drawing indicating the initiation and propagation of cracks on the MOS after five loading cycles, respectively, (**b**,**c**) microcracking along the interfaces between the 2nd-order lamellae in the outer layer after first and second loading cycles, (**f**,**g**) channel cracking in the inner layer after third and fourth cycles, (**I**,**j**) a crack propagating along the interface between the inner and middle layers, and (**k**,**l**) an overall view of failed sample after the fifth loading cycle.

As shown in Fig. 3(a,b), multiple microcracks are developed along the interfaces between the 2nd-order lamellae in the outer and middle layers after the first cycle of loading up to 45 MPa, and the number of microcracks increases with increasing load to 83 MPa, as shown in Fig. 3(c,d). After the second cycle, channel cracking along the 2nd-order lamellar interfaces in the inner layer is also developed, but arrested by the interface between the inner and middle layers (Fig. 3(e,f)). As the loading is further increased to 137 MPa, more parallel channel cracks are produced, and earlier channel cracks become enlarged, as shown in Fig. 3(g). Furthermore, some earlier channel cracks also start to extend along the interface between macrolayers with a zig-zag path, as shown in Fig. 3(h–j). After the fifth loading cycle, most interfaces of the 1st-order and 2nd-order lamellae in the middle layer fail, as shown in Fig. 3(k,l). Furthermore, the fifth loading, which could only reach basically the same stress as that of the forth loading cycle (i.e., ~138 MPa), leads to a catastrophic failure (Fig. 3(k,l)). It is of interest to note that the macrocrack in the middle layer propagates along an oblique direction, which is a common phenomenon in this structure. For example, Menig *et al*.^22^ observed that the crack deflection also happened in the middle layer of *Strombus gigas* shell samples during compression, as the organic interfacial layers could arrest and deflect cracking when the loading direction was perpendicular to the 1st-order lamellae. On the other hand, both interfaces between macrolayers (i.e., inner/middle layer interface, and outer/middle layer interface) partly failed after the 4th cycle (Fig. 3(h–j)), which caused that the middle layer undergoes the majority of loading in the 5th cycle. Thus, the shear movement is relatively easier in the middle layer in this case.

It should be noted that the Young’s modulus or slope on the stress-strain curve for the fourth loading cycle changes from 6.2 GPa to 5.0 GPa, reflecting a significant damage to the microstructure by the crack propagation along the interface between the inner and middle layers to the bottom of the sample (Fig. 3(i)). This suggests that the lower part of the inner layer has been broken from the whole sample. However, it is interesting to note that in the subsequent fifth loading cycle the Young’s modulus has reached a similar value to that of the previous three loading cycles. This indicates that the remaining middle and outer layers can still bear the load until the middle layer fails (Fig. 3(l)), even though the inner layer has been broken.

### Crack bridging

**Figure 4:**
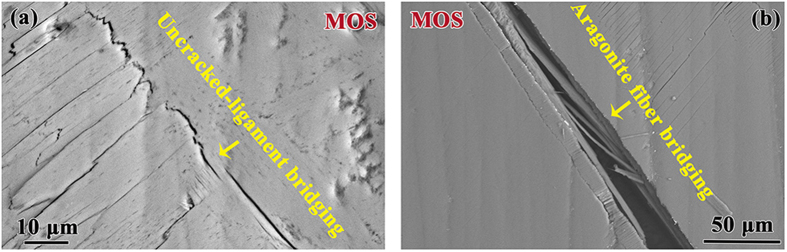
(**a**) Uncracked-ligament bridging and (**b**) aragonite fiber bridging in the crossed-lamellar structure.

During the propagation of channel cracks, two major types of bridging are observed on MOS. Uncracked-ligament bridges appear on the propagation paths of cracks (as indicated in Fig. 4(a)), along with crack deflection/twist in the form of zig-zag paths and multiple microcracks perpendicular to the main crack. Inside the crack aragonite fibers are stretched across the crack but still connected to both sides, forming the bridges (Fig. 4(b)). The crack bridging phenomenon has also been observed to be present in human bone^1^,^4^, *Alligator osteoderms*^31^, *Leatherback sea turtle* shell^32^, etc. The bioinspired materials including bio-inspired glass^13^,^14^, bio-inspired ceramic-based composites^12^, and bio-inspired (“nacre-like”) hybrid polymer composites^33^ also inherit these bridging mechanisms during deformation.

### Fracture surface characteristics

**Figure 5:**
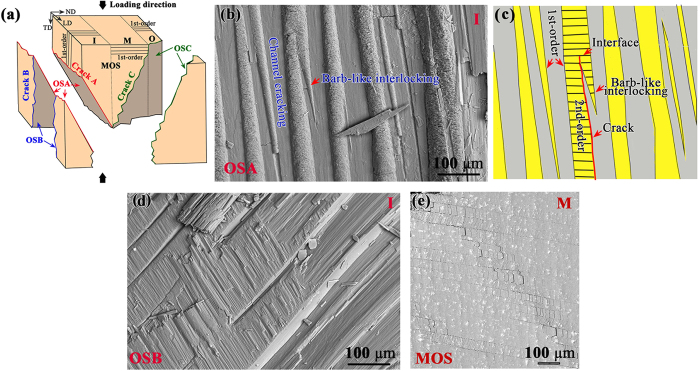
(**a**) Schematic drawing representing a failed sample after compressive loading and different observational directions, (**b**) channel cracking and barb-like interlocking observed on OSA, (**c**) schematic drawing of a barb-like interlock and its interaction with a crack, (**d**) broken fibers left on the surface of adjacent intercrossed lamella, and (**e**) interfaces to be sufficiently weakened before the failure of the inner layer.

After the stepwise loading-unloading compression tests, the sample was divided into three parts by three main cracks along different directions, as shown in Fig. 3(l) which is schematically re-plotted in Fig. 5(a). The fracture surface characteristics of each crack in Fig. 5(a) is carefully examined, where the observational surfaces (OS) are referred to as OSA, OSB, and OSC for cracks A, B, and C, respectively. On the OSA one can see that the shape of the 1st-order lamella in this sea shell is basically irregular (Fig. 5(b)). It is of particular interest to observe a unique phenomenon of interlocking in some sheets of the 1st-order lamellae, as indicated by the red arrows in Fig. 5(b). It appears that some neighboring 1st-order lamellae closely “bite” each other by a kind of special structure. Almagro *et al*.^20^ described this kind structure as a cone extending from the 1st-order lamellae. This type of fishhook barb-like interlocks is different from the “platelet interlock” observed in nacre^34^,^35^,^36^. When a crack propagates along the interface into the barb-like interlocks, the 2nd-order lamellar perpendicular to the crack propagation direction may arrest the propagation until all fibers in the barb-like structure are failed, as indicated by the schematic in Fig. 5(c). Figure 5(d) shows the fractured fibers left on the neighboring sheet, and it can easily be imagined that the bundle of fibers drag the upper lamellae until the complete failure. This type of interlocking plays an important role in arresting the main crack propagation, as such an interlock needs to be broken or yielded before the complete transfer of loads to the boundary between two adjacent sheets, hence increasing the toughness of materials.

Furthermore, the irregular shape of the 1st-order lamellae may lead to general hardening. As the sheets are pushed, the tumid parts of a sheet tend to expand more towards the left and right adjacent intercrossed sheets, generating a compressive effect in that region. The transverse compressive stress is balanced by transverse tensile stresses in other regions. The transverse tensile stresses are magnified by stress concentrations at the tumid regions, which could lead to sheet delamination and release the locking mechanism. General hardening is necessary for the sheet gliding when spreading from local regions to the whole volume with increasing applied load, which causes all the interfaces to be sufficiently weakened until the failure, as shown in Fig. 5(e). If the interfaces are straight, no hardening would be generated and a fast and catastrophic propagation of cracks would happen. As a result, this type of irregular interface structure spreading throughout the volume of the crossed-lamellar structure results in a further enhancement in the strength and fracture toughness of the sea shell.

**Figure 6:**
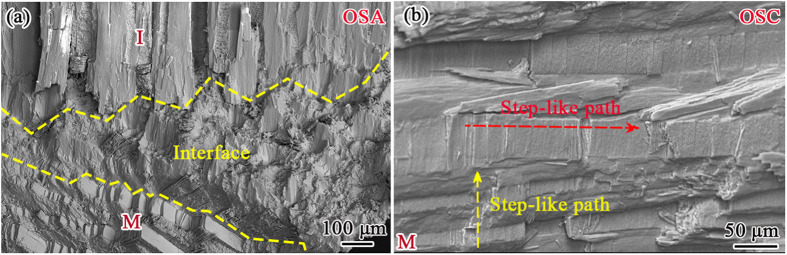
(**a**) Morphology of the interface between the inner and middle layers, and (**b**) step-like propagating path in the middle layer.

From the stepwise compressive tests in Fig. 3, it has been seen that the interface between the inner and middle layers can effectively arrest the propagation of channel cracks, since the interface exhibits a complicated structure, as shown in Fig. 6(a). Even though the crack begins to propagate along the interface when the load is high enough, it is easily deflected due to the complicated structure, leading to a zig-zag path, as shown in Fig. 3(i,j). After the interface fails, the crack will go into the middle layer, which shows step-like paths not only inside individual 1st-order lamellae but also between the adjacent 1st-order lamellae along the stacked direction, as shown in Fig. 6(b). The above observed barb-like interlocks, irregular shape of 1st-order lamellae and complicated interfacial structure between macrolayers play an important role in toughening and strengthening the present *C. nobilis* shell.

## Discussion

**Figure 7:**
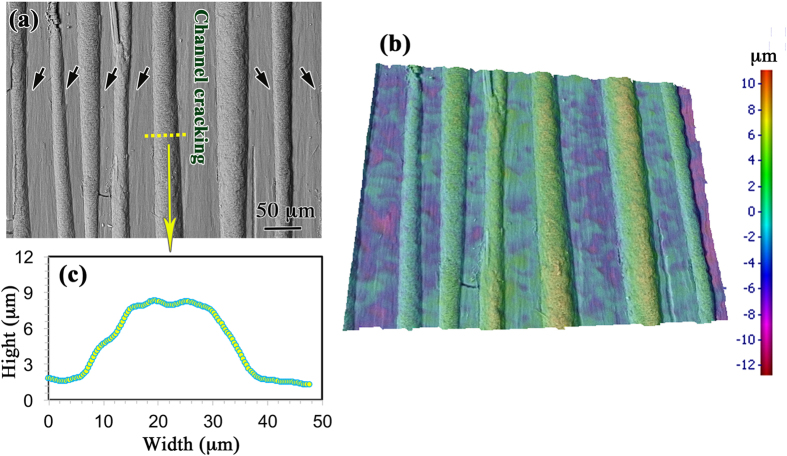
(**a**) A characteristic “convex” fracture surface morphology, (**b**) the corresponding 3D morphology, and (**c**) a representative height profile across the “convex” tougher lamella.

It is known that the building blocks or the 1st-order lamellae in the crossed-lamellar structure are oriented either in a “weak” orientation or “tough” orientation with respect to a given loading direction. In the present work the tiled direction of the 1st-order lamellae in the inner layer is nearly parallel to the loading direction, and the interfaces of the 2nd-order laths inside some 1st-order lamellae have an angle of ~33° to the compressive loading direction, which become the weak lamellae along with the neighboring tough intercrossed lamellae. Such a weak-tough alternate arrangement results in a third type of energy dissipation mechanism, as shown in Fig. 7(a,b) where the cracking across the tough lamellae exhibits a convex path as revealed from the height profile shown in Fig. 7(c). Such a characteristic fracture morphology has not been reported in the literature so far, to the best of our knowledge. The average starting angle *θ* from the weak lamellae forming flat channel cracks to the adjacent tough lamellae with a curved/convex fracture morphology is observed to be ~50°. The convex fracture morphology could be approximately considered as part of a lying cylinder with several micrometers in height protruding from the plane of channel cracks. The area per unit length, *S*, of this partial cylinder could be calculated by the following equation,


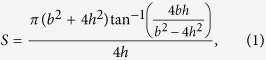


where *b* and *h* is the width and height of “convex” profile of tough lamellae, respectively. Then, an average value of ~39 μm^2^ for *S* is obtained, which is about 20% higher than that of the flat plane. It follows that the “convex” fracture surface in the tough lamellae would absorb ~20% more energy, resulting in a higher toughness.

**Figure 8:**
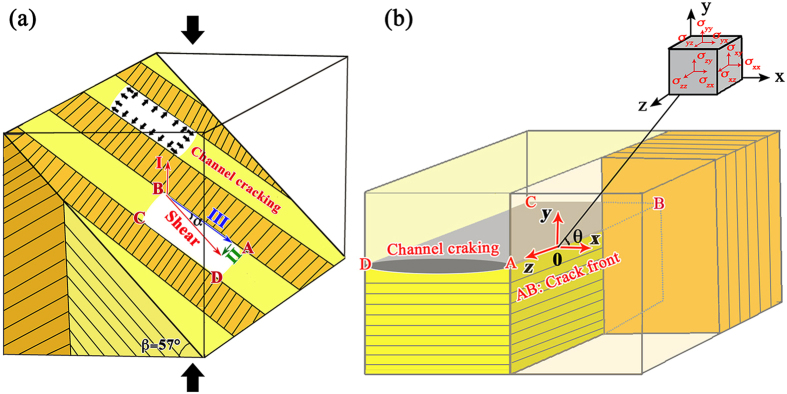
(**a**) Schematic diagram for channel cracking in the inner layer, and (**b**) the distribution of stress field at the interface between two 1st-order adjacent lamellae ahead of the crack tip.

Linear elastic fracture mechanics is used to analyze the formation of the above “convex” fracture surface by considering all three loading modes. Here the slightly simplified plane strain configuration is modeled by a two-lamellar composite, and the tough and weak lamellae are assumed to have the same Young’s modulus *E* and Poisson’s ratio *ν* (with a value of 0.3^23^,^28^). During compressive deformation, the applied load is transmitted through the material by compressive normal stress (Mode I) and shear stresses at the interfaces. The shear stress can be further resolved into Mode II in-plane shear stress and Mode III outer-of-plane shear stress with respect to the tough lamellar sheet, as shown in Fig. 8(a). When the applied load increases, these stresses will increase accordingly until one possible failure mode is activated. Some channel cracks along the 2nd-order interfaces in the weak lamellae are first developed at some positions with defects (such as growth defects and containing impurities^2^,^23^). For an initiated channel crack ABCD shown in Fig. 8(a), the crack front AD and BC would extend under compressive loading. As the applied load further increases, the crack front AB and CD would extend as well, where a likely initiation associated with the extension of the channel crack into the tough lamellae will be reached. This could be understood and roughly analyzed using a linear elastic finite-element analysis model^37^. As the crack front AB would be subjected to both normal stress (Mode I) and shear stress (Modes II and III), the stress field ahead of the crack tip AB (Fig. 8(b)) could be expressed as^38^,


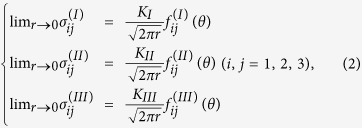


where *K*_*I*_, *K*_*II*_ and *K*_*III*_ are the stress intensity factors for Modes I, II and III, respectively. *f*_ij_(*θ*) is a function of crack deflection angle *θ*. Under a uniaxial loading, the stress intensity factors for a crack oriented at an angle of *β* = 57° are given by,


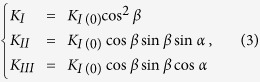


where *K*_I(0)_ is the Mode I stress intensity factor when *β* = 0°, and *α* is the angle between the overall shear stress direction and the interface between the weak and tough lamellae (Fig. 8(a)). The three principal stresses ahead of the crack tip can be calculated by the follow equation,


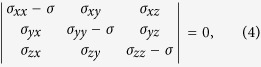


where the results are described as follows,


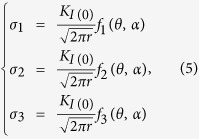


As seen from Figs 3(l), 5(a) and 7(a), this shell sample is sheared to fracture. Thus, the theory considered here to model crack growth direction is based on the shear stress maximization^38^, i.e.,


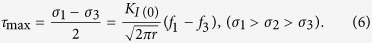


**Figure 9:**
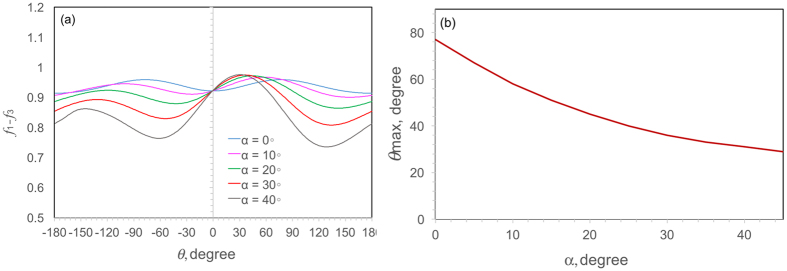
(**a**) *f(θ*) as a function of *θ* with varying angles of *α*, and (**b**) the change of *θ*_max_ with angle *α*.

Based on the above formulation, Fig. 9(a) shows the distribution of *f(θ*) = *f*_1_ − *f*_3_ as a function of *θ* with varying angles of *α*. When *α* = 0° (i.e., the resolved shear stress is just along the interface border line, Fig. 8(a)), the in-plane Mode II shear stress is zero, which means that the crack front is subjected to mixed Modes I and III. Under such a mixed mode, the deflection angle *θ* of crack extension becomes the maximum (~77°). Figure 9(b) shows the value of *θ*_max_ corresponding to the maximum value of *f(θ*) as a function of angle *α*. It is seen that *θ*_max_ decreases with increasing *α*. This suggests that the presence of Mode II in this condition weakens the deflection angle of crack extension towards the tough lamellae. In a *Strombus gigas* shell subjected to four-point bending^23^, both Modes I and II were considered to model the large scale bridging, although the fracture surfaces exhibited a flat feature. The special arrangement of the crossed-lamellar structure under compression can provide a more complicated stress field ahead of the crack tip even if samples are deformed in a uniaxial loading condition, which can cause an obvious deflection at the crack tip and hence increase the toughness of materials.

As analyzed above, the criterion of shear stress maximization could be used to estimate the deflection of cracks in the tough lamellae fairly accurately, while it was normally applied to the ductile materials. One possible reason could be that a certain extent of plastic deformation is present in the local portion around the crack tip. Li *et al*.^39^,^40^ reported that the individual 3rd-order lamellae of the crossed-lamellar structure consist of both aragonite nanoparticles and biopolymers, and the nanoparticle rotation effectively dissipates energy through plastic deformation in individual lamellae by nanoscale three-point bending tests on individual 3rd-order lamellae. Ultimately, cracks tend to propagate along the maximum shear stress at the crack tip since the local area exhibits a certain degree of plastic deformation.

One could also consider that the crossed-lamellar structure is composed of two kinds of substrates bonded together with interfaces. The weak lamellae could be seen as matrix materials, and the tough lamellae as fiber inclusions. Let the channel cracks to be developed inside the matrix, then the deflection criterion at the fiber/matrix interface can be expressed as^41^,


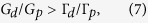


where *G*_d_ and *Γ*_d_ represent the energy release rate and surface energy in the case of deflection, respectively, and *G*_p_ and *Γ*_p_ refer to the corresponding quantities in the case of penetration. The composite can be tough if the fibers remain intact and the interface is weak enough for the matrix crack to be deflected along the interface. Otherwise, the matrix crack would penetrate into the fibers and thus the composite is brittle like a mono-lithic ceramic^42^. Mirkhalaf *et al*.^14^ proposed a new criterion which is related to the crack deflection angle *θ* as follows,


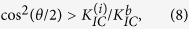


where *K*_IC_^(i)^ and *K*_IC_^(b)^ are the critical stress intensity factors (fracture toughness) of the interface and the bulk of the material, respectively. In the buck glass^14^, if the crack propagates along the interface, their experiments show that interface could deflect the crack up to an angle of *θ* = 60°, similar to the result observed in the present shell. The apparent toughness increases with increasing angle from half of the toughness of glass at *θ* = 0° up to the toughness of glass at *θ* = 70°, which is in agreement with the result discussed on the basis of linear elastic fracture mechanics above. In the *C. nobilis* shell, fiber failure is suppressed at the matrix crack front, and the crack deflection results in a pullout effect with a convex morphology (Fig. 7), which would contribute to the toughness of materials^43^.

It can be concluded that the complicated architecture of this *C. nobilis* shell enhances its strength and toughness by invoking several energy-dissipating mechanisms: microcracking at lower loads, channel cracking in the weak lamellae, crack bridging and crack deflection in the tough lamellae at higher loads. The superior mechanical properties of the crossed-lamellar structure are strongly related to its distinctive structural arrangement with complex interfaces, which can provide complex stress field at the crack tip to drive crack deflection and improve the toughness of materials. The crossed-lamellar microarchitecture in the present *C. nobilis* sea shell can thus be used to guide the bio-mimetic design of tougher and stronger materials.

## Materials and Methods

The target material in the present study is *C. nobilis* shell, which belongs to the Gastropoda class. This shell, also known as *Noble Volute*, has a logarithmic spiral shape. In the present work, all the analyses were based on the principal directions LD, TD and ND, where LD stands for the longitudinal direction (i.e., the shell axis direction, meaning the spiral axis direction), TD indicates the transverse direction (i.e., the growth direction), and ND denotes the normal direction (i.e., the shell thickness direction), as indicated in Fig. 1(a). The sample for the initial microstructural examinations was cut via a slow diamond saw and then directly broken using a plier. The hierarchical structures present on the TD-ND fractured surface were observed via a scanning electron microscope (SEM, JSM-6380LV) equipped with three-dimensional (3D) surface/fractographic analysis capacity. A rectangular X-ray diffraction (XRD) specimen of approximately 15 mm × 10 mm was cut and ground up to 600 grit sand paper in three steps to specifically expose the inner, middle and outer layers of the sea shell, respectively. Attempt was made to achieve the middle of each macroscopic layer, then XRD analysis was conducted in each step via a Panalytical X-ray diffractometer using Cu *K*_*α*_ radiation (wavelength *λ* = 0.15406 nm) at 45 kV and 40 mA.

Compression tests were conducted at room temperature with a constant strain rate of 1.0 × 10^−4^ s^−1^, and the loading direction is along TD. Orthorhombic compressive specimens with a dimension of approximately 4.0 mm (LD) × 4.0 mm (TD) × 5.0 mm (ND) were prepared. A special sample holder was machined to facilitate the specimen preparation, aiming to ensure the surfaces in contact with the compressive plates to be parallel. In order to observe crack initiation and propagation characteristics during deformation, stepwise compressive tests were also performed on some samples with a TD-ND surface carefully polished using diamond paste down to 1 μm. In evaluating the compressive stress-strain curves, the machine deformation was eliminated using a calibration curve to obtain the actual deformation amount of test samples. The obtained compression strength *σ* was analyzed via a two-parameter Weibull distribution^44^,^45^:


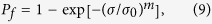


where *P*_*f*_ is the failure probability, *m* is the Weibull modulus, and *σ*_o_ is the characteristic strength.

## Additional Information

**How to cite this article**: Ji, H. *et al*. *Cymbiola nobilis* shell: Toughening mechanisms in a crossed-lamellar structure. *Sci. Rep.* **7**, 40043; doi: 10.1038/srep40043 (2017).

**Publisher's note:** Springer Nature remains neutral with regard to jurisdictional claims in published maps and institutional affiliations.
